# Implementation of a neonatal hepatitis B immunization program in rural Karenni State, Myanmar: A mixed-methods study

**DOI:** 10.1371/journal.pone.0261470

**Published:** 2021-12-20

**Authors:** T. Hugh Guan, Hnin Nandar Htut, Colleen M. Davison, Shruti Sebastian, Susan Andrea Bartels, Soe Moe Aung, Eva Purkey

**Affiliations:** 1 Department of Family Medicine, Queen’s University, Kingston, Ontario, Canada; 2 B.K.Kee Foundation, University Avenue Road, Bahan Township, Yangon, Myanmar; 3 Department of Public Health Sciences, Faculty of Health Sciences, Queen’s University, Kingston, Ontario, Canada; 4 Division of Clinical Sciences, Northern Ontario School of Medicine, Mindemoya, Ontario, Canada; 5 Department of Emergency Medicine, Queen’s University, Ontario, Canada; 6 Civil Health and Development Network, Loikaw, Karenni State, Myanmar; University of Cape Town, SOUTH AFRICA

## Abstract

**Background:**

Hepatitis B infection is a major health concern in Myanmar. Hepatitis B birth dose vaccination to prevent mother-to-child transmission is not universal, especially in births outside of health care facilities. Little is documented about delivery of immunization programs in rural Myanmar or in conflict-affected regions. To address this gap, this study describes the implementation of a novel community delivered neonatal hepatitis B immunization program in rural Karenni State, Myanmar.

**Methods:**

A mixed-methods study assessed the effectiveness and feasibility of hepatitis B birth dose immunization program. 1000 pregnant women were screened for hepatitis B virus (HBV) infection using point of care testing. Neonates of HBV positive mothers were immunized with a three dose HBV vaccine schedule at birth, 1, and 6 months of age. HBV testing was completed for children at 9 months to assess for infection. Descriptive statistics were collected including demographic data of mothers, neonatal vaccination schedule completion, and child HBV positivity at 9 months. Qualitative data examining barriers to implementation were collected through semi-structured interviews, participant-observation, and analysis of program documents. Themes were codified and mapped onto the Consolidated Framework for Implementation Research.

**Results:**

46 pregnant women tested HBV positive leading to 40 live births. 39 women-child dyads were followed until the 9-month age mark. With the exception of two neonates who received their birth dose past 24 hours, all children received their vaccines on time. None of the 39 children tested positive for HBV at nine months. Themes regarding barriers included adaptability of the program to the rural setting, friction with other stakeholders and not meeting all needs of the community. Identified strengths included good communication and leadership within the implementing ethnic health organization.

**Conclusion:**

A community delivered neonatal HBV vaccination program by ethnic health organizations is feasible and effective in rural Myanmar.

## Introduction

The hepatitis B virus (HBV) is a blood-borne pathogen that infects the liver and is a cause of global morbidity and mortality with an estimated 257 million people living with chronic HBV infection in 2015 [1]. Infection can cause acute hepatitis or lead to chronic infection resulting in liver cirrhosis and hepatocellular carcinoma. Within the World Health Organization’s (WHO) Southeast Asian Region, Myanmar has the highest rates of perinatal transmission [2]. There is an estimated 16 perinatal chronic HBV infections per 1000 live births in Myanmar and an estimated 6.5% of the population in Myanmar has chronic HBV [2, 3]. There is also an estimated 3.8% of 5 year old children in Myanmar who are hepatitis B surface antigen (HBsAg) positive [2]. Further data from the Thai-Myanmar border established a HBV seroprevalence of 8.5% among pregnant women whom were primarily ethnic Karenni [4]. Given the high prevalence of HBV infection, high population health burden is likely. Cirrhosis of the liver, a long-term consequence of HBV infection was estimated to be the fifth leading cause of death in Myanmar in 2017 [5].

The prevention of mother to child transmission (PMTCT) of HBV is important as the likelihood of developing chronic hepatitis B is estimated to be as high as 90% among those infected at birth, and between 15 and 40% of those with chronic infections will progress to develop cirrhosis or hepatocellular carcinoma [6, 7]. Rates of vertical transmission vary significantly depending on the characteristics of maternal infection, ranging from 10–40% with positive maternal HBsAg and negative maternal hepatitis B e antigen (HBeAg) to as high as 70–90% if both HBsAg and HBeAg are positive [6, 7]. Although maternal screening programs and universal vaccination of newborns (birth dose) have reduced HBV vertical transmission rates worldwide, at present such programs are not consistently available in Myanmar. HBV vaccination, HBV birth dose, as well as potential use of antiviral therapy from 28 weeks gestation to 3 months postpartum is part of Myanmar’s central governmental health policy, but implementation is not complete in remote regions, and this situation is likely to worsen with the February 2021 military coup [2, 3, 8]. This may be due to the lack of governmental health facilities in rural regions and limited accessibility. Travel times to governmental facilities are on average longer than to ethnic and community based health service providers [9]. In Myanmar, birth dose HBV vaccination is mainly provided for institutional deliveries in hospitals, and was introduced in 2016 [2]. Although Myanmar has an estimated three dose HBV vaccine coverage of 90%, only 17% of the population receive a HBV vaccination series at birth in 2019, up from 7% in 2018 [10]. Additionally, only 36% of births in Myanmar occur in health facilities, creating a gap in the delivery of HBV birth dose vaccination [2].

The combination of hepatitis B immunoglobulin (HBIG), birth dose vaccine, and three dose vaccine series is estimated to reduce the vertical transmission rate to 3% [6]. Within the last few years, there are also recommendations for the use of peripartum antivirals in addition to vaccine and HBIG for decreasing vertical transmission and this is the formal recommendation of the WHO as of 2020 [11]. However, the access to WHO recommended therapy is not always possible in resource limited settings [11]. Therefore, further studies assessing feasible regimens in resource limited settings where testing and treatment are a challenge may be required. A 2015 meta-analysis showed no significant difference in HBV infection or in sero-protection markers between birth dose vaccine alone versus vaccine plus HBIG for HBsAg+/HBeAg- mothers [12]. A 2017 Chinese study showed no differences in perinatal transmission between birth dose vaccine alone versus vaccine plus HBIG [13]. However, both of these studies were conducted among women who were HBsAg + / HBeAg-. A third study in rural India, which screened only for HBsAg positivity, found that vaccine alone had an effectiveness of only 64% in a non-study setting [14].

Little is documented about the factors that may influence HBV birth dose immunization program implementation in a rural, resource limited, and culturally variable Myanmar setting. Several studies illustrate HBV immunization programs in rural locales within surrounding Asian countries, but no studies have been conducted in Myanmar. A study on HBV immunization in rural South India described implementation and immunization rates [14], while another study in rural China explore vaccination rates, vaccine awareness, and serology to measure out-of-cold-chain vaccine success [15]. Additional studies in rural Laos and Indonesia also investigated out-of-cold-chain HBV vaccination and barriers to providing the birth dose of HBV vaccine respectively [16, 17]. The present study adds to the existing literature on HBV immunization implementation in a rural setting within Asia.

Myanmar presents a unique opportunity to proactively integrate context and implementation science into program design to create culturally aware and specific sustainable interventions that may grow with the health system over time and engage all stakeholders. Using the Myanmar context and existing definitions for evaluating program implementation, this study describes a targeted HBV vaccination program for PMTCT in a rural region and assesses the feasibility of such a program in a resource limited setting.

## Methods

A mixed methods analysis was completed. Based on the framework of Palinkas et al. (2011), a mixed method design in an implementation study [18] allows for evaluation of both process and outcomes. Additionally, mixed methods analysis allows exploration of the content as well as the context of the intervention [18]. As this study appears to be the first describing a HBV immunization program in a conflict-affected rural region in Myanmar, combining quantitative and qualitative data provides a more complete picture of implementation and increases the rigor of the evidence through triangulation [19].

### Program description

In 2016, a pragmatic field test case study in Karenni and Shan states, Myanmar implemented a targeted HBV vaccination program for PMTCT in rural areas. The HBV vaccination program was a collaboration between three partners: the Civil Health and Development Network (CHDN) of Karenni State, Myanmar, B.K.Kee Foundation, an American non-governmental organization operating out of Yangon, Myanmar, and Queen’s University in Kingston, Canada. CHDN is a network bringing together six ethnic health organizations to provide access to quality health care services for the communities in Karenni State. Its mission is to deliver healthcare services for populations where government services are limited or nonexistent. The vaccination program was funded by B.K.Kee Foundation, and technical and financial support for the monitoring and evaluation components was provided by Queen’s University.

**Fig 1:**
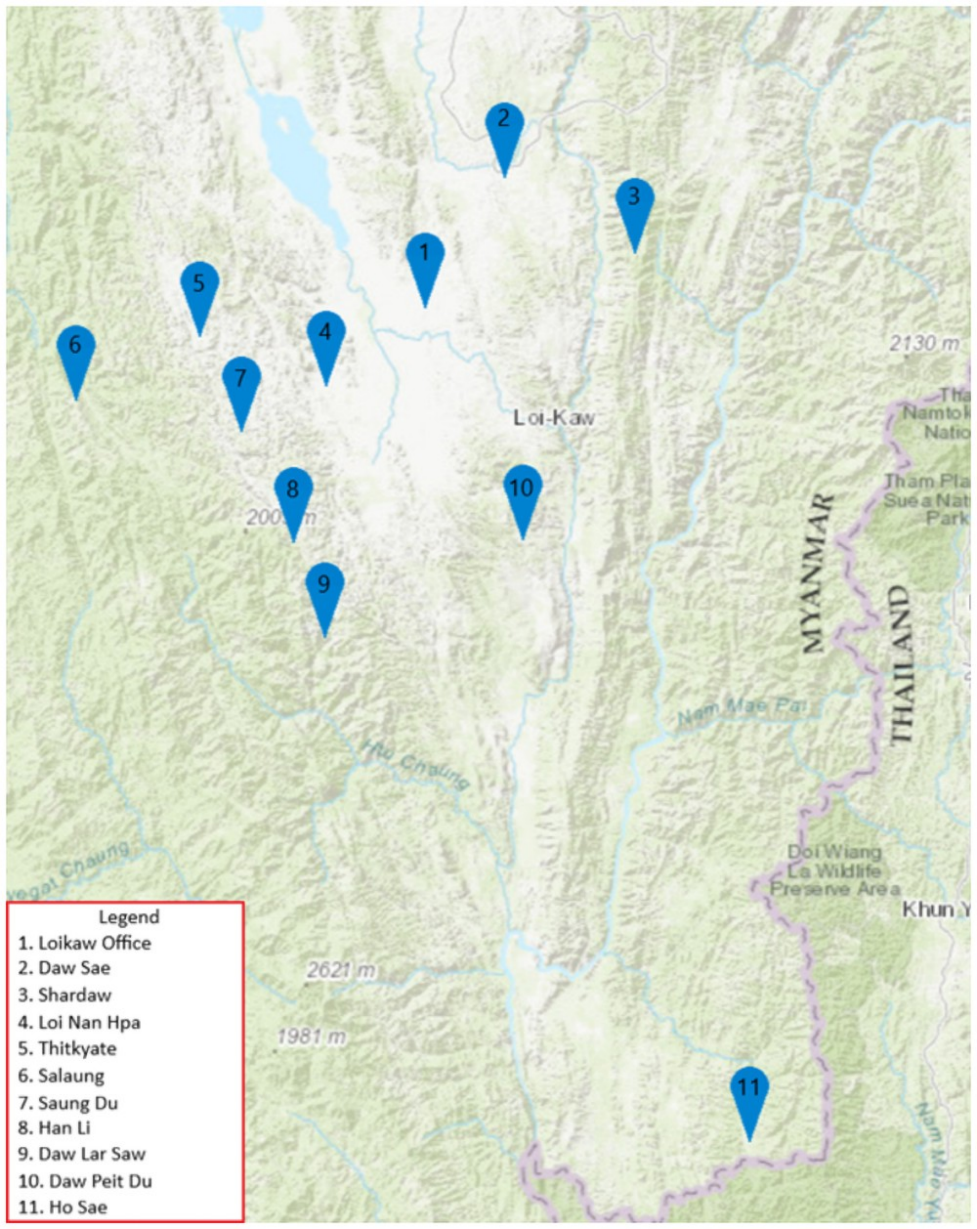
Map of CHDN main office and clinic locations. Map courtesy of U.S. Geological Survey.

From January 2016 to February 2017, one thousand pregnant women were recruited sequentially and screened for HBV with the ABON Hepatitis B Surface Antigen Rapid Test Device. The relative sensitivity and specificity of these devices are 99.8% and 99.2% respectively [20]. Verbal informed consent was given by the women for both HBV screening and HBV vaccination for their newborns. Recruitment occurred in ten villages under the jurisdiction of CHDN in Karenni (nine villages) and Shan (one village) states, with a catchment population of 144,000 (Fig 1). Within this catchment area, there were an estimated 9400 women of reproductive age [21]. These villages are small, and under normal circumstances, the majority of women in these villages would be followed by Community Health Workers (CHWs) from CHDN during their pregnancies, and therefore recruitment occurred at routine antenatal visits. CHWs provide routine prenatal care, as well as screening and treatment for other illnesses, and child wellness checks among others. In many settings they are the only healthcare providers available within a reasonable travel distance. Women who screened positive for HBsAg continued to be followed by CHWs at clinic check ups and home visits until the birth of their children as would normally occur throughout their pregnancy. A three-dose vaccination schedule was implemented for newborns of HBsAg positive mothers, with the first dose scheduled to be given within the first 24 hours of birth, the second at one month, and the final dose at six months. Individualized systems were put in place to notify CHDN workers of the need for the birth dose, depending on where the woman delivered (at home, at a CHDN clinic, or in a government or private hospital). Specific recommendations around location of delivery did not depend on HBsAg status. Generally, contact was made by cellphone by a family member if the birth was unattended, or by a health worker if the birth was attended. This occasionally required travel by the person making the call due to unreliable cellphone service in certain villages. ABON HBsAg rapid tests were completed at 9 months of age to assess for HBV infection in the children.

Vaccines were stored in and delivered from the CHDN main office due to a lack of reliable electricity and/or refrigerators in rural clinics and the presence of a backup generator in the main office. Vaccine refrigerators were monitored with twice daily temperature checks for optimal temperatures between 2 to 8°C. CHWs at the clinics contacted the main office when vaccine was required and these vaccines were then delivered to each clinic. The cold chain was maintained during transport through cold boxes adapted following WHO recommendations [22].

Training of clinic staff was completed in a two-day pre-implementation workshop, and monitoring of study progress and data collection was undertaken through field visits to each clinic one month prior to the start of recruitment and nine months into the program. Training included information about the theory behind the program (information on HBV, why vaccination would be important, etc.), standardized program rollout, and effective maintenance of data logbooks. The follow-up nine-month field visit assessed the implementation of the program through the collection of quantitative and qualitative data. All clinics were supported by the CHDN central office which also provided the day-to-day and field supervision during implementation.

### Data collection and analysis

Quantitative data were collected using clinic logbooks and qualitative data were obtained through in depth interviews, analysis of textual data in the clinic logbooks and through field notes from participant observation.

Demographic information and HBV screening results for mothers and children were collected by CHDN staff and recorded in a Microsoft Access Database. Data were analyzed with SAS Enterprise version 7.1. Descriptive statistics were completed for all screened pregnant women and their newborns.

Qualitative data was collected through semi-structured interviews, examination of monitoring and evaluation documents, and the recording of field notes from participant-observation. The multi-modal approach allowed for methodological triangulation to increase the rigor of results. A theoretical approach of critical realism was used for the qualitative analysis. Critical realism describes how observed phenomena can approximate explanatory mechanisms through a lens of shared meaning making [23].

Nine semi-structured interviews with CHDN and B.K.Kee staff were conducted nine months after implementation. Purposeful sampling was conducted selecting individuals involved in the intervention and study administration and implementation. Participants included CHDN front-line CHWs, CHDN central office staff including managers and supervisors, and B.K.Kee program management. An interview guide was used focusing on process, barriers and facilitators of implementation. A translator was used to conduct eight out of the nine interviews in Kayah and the ninth was conducted in English. Interviews were recorded and transcribed with direct translations to English where needed. Transcriptions were coded in NVivo version 11 using thematic analysis along the methodology outlined by Braune and Clarke [24].

Focused participant-observation was also completed during the two field visits–the first just prior to implementation (during and immediately after training), and the second, nine months after initial implementation of the study. Two researchers took notes during the first field visit and developed consensus upon thematic areas, and one of the original researchers continued observations of clinics in the second field visit.

A second layer of analysis involved mapping data from the in-depth interviews, participant observations, and the examination of monitoring and evaluation documents onto the Consolidated Framework for Implementation Research (CFIR) [25]. It was decided *a priori* that observations and interviews would focus on mapping barriers and challenges in program implementation within the resource limited, conflict-affected setting rather than focusing on all aspects of the broader implementation experience. A focus on barriers and challenges allowed for resources to be dedicated towards understanding areas of program improvement. CFIR is a framework developed through consolidation of existing implementation theories which allows researchers to assess interventions across five major domains (intervention characteristics, outer setting, inner setting, characteristics of individuals, and process) [25]. These domains, and the constructs that underpin them, provide a framework against which an intervention or innovation can be examined and evaluated. CFIR was used as a comprehensive meta-theoretical framework to examine the implementation of the HBV vaccination program.

Local leaders at B.K.Kee Foundation and CHDN reviewed and approved the study protocol for the monitoring and evaluation component of this project. It is noteworthy that the testing and vaccination components of the project were planned prior to the involvement of Queen’s University as part of CHDN health services delivery programs. Research ethics for this study was obtained through the Queen’s University Health Sciences and Affiliated Teaching Hospitals Research Ethics Board. Due to low levels of trust between ethnic health organizations (in this case CHDN) and the central Myanmar government, as well as a history in which CHDN operates its health programs independently of the central Myanmar government, an explicit decision was made not to seek local Myanmar ethics approval in collaboration with local partners. Instead, written consent and support for the study was obtained from CHDN and B.K.Kee Foundation, and, retrospectively, the newly formed Community Ethics Advisory Board, operating out of Mae Sot, Thailand, with representation from ethnic health organizations, was consulted on the project. Analyses and perceptions were influenced by the fact that the majority of the researchers were trained in the Western scientific and biomedical traditions. It is also acknowledged that the researchers are in a position of authority given the relative social and economic differences between the researchers and the implementers and participants of this study. The differences in cultural backgrounds and unequal power dynamics between researchers and participants can contribute to a certain interpretation of the results, which may not be inclusive of all other perspectives [26]. To ensure credibility of the data and analysis, both B.K.Kee and CHDN were involved throughout the research process including project design (led by B.K.Kee and CHDN), ethics submission, the review of all reports, conference publications and presentations, and all published materials. Likewise, retrospectively, a newly formed Community Ethics Advisory Board in Mae Sot, Thailand, with representation from local ethnic health organizations and community partners, was consulted on this work.

## Results

### Sociodemographic data and participant outcomes

Demographic information for the 1000 pregnant women screened are summarized in Table 1. The median age was 27 years old with a median of 4 people living in the same household. The majority of the women were married, identified subjectively as middle income, and were farmers.

**Table 1:** Demographics of pregnant women screened for HBsAg.

Variable	Category	All Women (n = 1000)	HBV Infected (n = 46)
Age (Years)	Median (Range)	27 (16–51)	25 (18–44)
# of People in Household	Median (Range)	4 (1–15)	4 (2–8)
Socioeconomic Status—Subjective report	Rich (%)	18 (1.8)	1 (2.2)
	Middle (%)	719 (71.9)	38 (82.6)
	Poor (%)	263 (26.3)	7 (15.2)
Married	Yes (%)	995 (99.5)	46(100.0)
	No (%)	5 (0.5)	0 (0)
Occupation	Farmer (%)	971 (97.1)	46 (100)
	Teacher (%)	8 (0.8)	0 (0)
	Shopkeeper (%)	4 (0.4)	0 (0)
	Other (%)	17 (1.7)	0 (0)

Out of 1000 women screened for HBV, 46 tested positive for HBsAg providing an estimated HBV prevalence of 4.6% among women of childbearing age in the catchment area. Of the 46 HBV positive women, 40 had live births, 3 had stillbirths, and 3 had spontaneous abortions. Of the 40 live births, 37 (92.5%) received their first dose of HBV vaccine within the first 24 hours of birth. Two newborns (5%) received the first dose, but not within the recommended first 24 hours. One (2.5%) newborn dropped out of the program before vaccination. The remaining 39 children (97.5%) received their second and third dose of HBV vaccine on schedule. All 39 children who remained in the program tested HBsAg negative at 9 months of age.

Of the 30 births with a recorded location, 17 (56.7%) were homebirths while 12 (40%) were at a hospital and 1 (3.3%) was at a clinic. Out of the homebirths, 2 (11.8%) did not receive their first dose within 24 hours. All hospital and clinic births had timely first doses.

Characteristics of the birth setting for children born to HBV positive mothers (n = 46) are provided in Table 2.

**Table 2:** Characteristics of birth settings.

Variable	Category	n (%)
Attendant (n = 24)*	Doctor	12 (50.0)
	Traditional Birth Attendant	3 (12.5)
	Community Health Worker	5 (20.8)
	Midwife	3 (12.5)
	Unattended	1 (4.2)
Setting of Delivery (n = 30)*	Home	17 (56.7)
	Hospital	12 (40.0)
	Clinic	1 (3.3)

### Pre-implementation training and community education

CHDN’s CHWs were responsible for providing opportunities for face-to-face contact with participants for patient education, counselling, testing, and vaccination. Administration of the program was based at the CHDN central office in the provincial capital of Karenni State, Loikaw. A train-the-trainer model was used with B.K.Kee and Queen’s University partners (physicians and epidemiologists) training CHWs from each of the 10 regional clinics and four members of CHDN’s central administrative team on the objectives, implementation, and monitoring of the project. These CHWs, with support from CHDN, were then responsible for program implementation and for training of the other clinic staff in their respective clinics.

In total, forty CHDN staff were trained within the first two months of the program with 39 of those being CHWs. CHWs held information and education sessions regarding HBV in their communities 78 times over the study period with a total of 1921 participants. These information sessions were held in a variety of settings including in clinics, homes, and community spaces. The median number of people attendees per session was 24 with a range of 1–99 people per session.

#### CFIR mapping (Table 3)

**Table 3:** Consolidated framework for implementation research theme mapping.

**I. Innovation Characteristics**		
A. Innovation Source	Definition: Perception of key stakeholders about whether the innovation is externally or internally developed.	At the time of training, the innovation seemed to be conceptually owned by both B.K.Kee and CHDN. It is not clear whether the initial impetus was one or the other, or if this was co-developed.
B. Evidence Strength & Quality	Definition: Stakeholders’ perceptions of the quality and validity of evidence supporting the belief that the innovation will have desired outcomes.	Not discussed
C. Relative Advantage	Definition: Stakeholders’ perception of the advantage of implementing the innovation versus an alternative solution.	Not discussed
D. Adaptability	Definition: The degree to which an innovation can be adapted, tailored, refined, or reinvented to meet local needs.	Occurred throughout implementation due to challenges such as lack of electricity (and thus refrigeration) in sites where this had been expected and difficulties related to communication and transportation. Major challenges included (1) communication of delivery (based on cell service, availability of phones; (2) timely transportation of vaccine (given rainy season, distance, quality of roads, time involved, etc.)
E. Trialability	Definition: The ability to test the innovation on a small scale in the organization, and to be able to reverse course (undo implementation) if warranted.	The immunization program was essentially the pilot of the intervention. The decision at the end of this pilot was that the innovation could be implemented successfully on a broader scale.
F. Complexity	Definition: Perceived difficulty of the innovation, reflected by duration, scope, radicalness, disruptiveness, centrality, and intricacy and number of steps required to implement.	Immunization and testing were well within CHWs scope of practice, however the degree to which there was an expectation of intervention documentation for the purposes of monitoring and evaluation was an additional burden for providers, as was the idea of a formal consent process, which had to be changed during implementation.
G. Design Quality & Packaging	Definition: Perceived excellence in how the innovation is bundled, presented, and assembled.	Not discussed
H. Cost	Definition: Costs of the innovation and costs associated with implementing the innovation including investment, supply, and opportunity costs.	Test kit and vaccine cost was not identified as a barrier. However, staff commented on the lengthy vaccine transport time to clinic.
**II. Outer Setting**		
A. Needs & Resources of Those Served by the Organization	Definition: The extent to which the needs of those served by the organization (e.g., patients), as well as barriers and facilitators to meet those needs, are accurately known and prioritized by the organization.	While the intervention met the needs of newborns, no treatment was available or funded for HBV positive mothers. Likewise, testing was not available for non-pregnant community members despite active community engagement in the areas of education related to HBV and increased community interest in testing.
B. Cosmopolitanism	Definition: The degree to which an organization is networked with other external organizations.	Lack of communication and networking with government health facilities leading to implementation challenges when participants delivered in hospital, suspicion, conflict between ethnic community health workers and hospital staff.
C. Peer Pressure	Definition: Mimetic or competitive pressure to implement an innovation, typically because most or other key peer or competing organizations have already implemented or are in a bid for a competitive edge.	HBV vaccination is supposed to be universally available in Myanmar through government healthcare services. However, this is not the case in rural and remote ethnic regions where vaccine may not be available and/or population may not access government facilities.
D. External Policy & Incentives	Definition: A broad construct that includes external strategies to spread innovations including policy and regulations (governmental or other central entity), external mandates, recommendations and guidelines, pay-for-performance, collaboratives, and public or benchmark reporting.	Not discussed
**III. Inner Setting**		
A. Structural Characteristics	Definition: The social architecture, age, maturity, and size of an organization.	CHDN is a longstanding ethnic healthcare umbrella organization. B.K.Kee is a relatively new NGO, but has access to international funding sources and support for implementation science.
B. Networks & Communications	Definition: The nature and quality of webs of social networks, and the nature and quality of formal and informal communications within an organization.	Good relationships between CHDN and the health clinics facilitated communication, implementation, monitoring and evaluation. CHDN was able to support CHWs to improve data collection and to trouble shoot throughout implementation.
C. Culture	Definition: Norms, values, and basic assumptions of a given organization.	All ethnic healthcare organizations have as their stated mandate to provide care to the local ethnic population and immunization was perceived to be within their mandate. However, CHDN CHWs identified written consent as an uncommon part of their process and thought it would be intimidating for staff and participants in the program.
D. Implementation Climate	Definition: The absorptive capacity for change, shared receptivity of involved individuals to an innovation, and the extent to which use of that innovation will be rewarded, supported, and expected within their organization. Includes: Perceived need for change. Compatibility with existing processes Relative priority Incentives and rewards Goals and feedback Learning climate	Challenges related to literacy and education levels of healthcare workers with respect to their ability to integrate training materials and transfer this knowledge to practice. Good support for implementationby CHDN staff on and ongoing and iterative basis. The prioritization of this initiative was questioned in the context of a comparatively low prevalence rate compared with national rates, and the ongoing presence of many other, possibly more pressing, active health concerns among the population.
E. Readiness for Implementation	Definition: Tangible and immediate indicators of organizational commitment to its decision to implement an innovation. Includes: Leadership engagement Available resources Access to knowledge and information	Good engagement and support by leadership, adequate personnel provided by CHDN, and adequate resources provided by B.K.Kee Foundation. Although there was an initial training session followed by outreach and re-training by CHDN staff, staff identified a need for additional training throughout the project to improve implementation.
**IV. Characteristics of Individuals**		
A. Knowledge & Beliefs about the Innovation	Definition: Individuals’ attitudes toward and value placed on the innovation, as well as familiarity with facts, truths, and principles related to the innovation.	No concerns were identified.
B. Self-efficacy	Definition: Individual belief in their own capabilities to execute courses of action to achieve implementation goals.	Improved over the course of implementation through support from CHDN central staff.
C. Individual Stage of Change	Definition: Characterization of the phase an individual is in, as s/he progresses toward skilled, enthusiastic, and sustained use of the innovation.	Not assessed.
D. Individual Identification with Organization	Definition: A broad construct related to how individuals perceive the organization, and their relationship and degree of commitment with that organization.	Not assessed.
**V. Process**		
A. Planning	Definition: The degree to which a scheme or method of behavior and tasks for implementing an innovation are developed in advance, and the quality of those schemes or methods.	Data collection tools developed in advance did not meet the needs of the frontline CHWs. Pre-implementation training session had limitations in knowledge transfer due to issues of language comprehension and hierarchy.
B. Engaging	Definition: Attracting and involving appropriate individuals in the implementation and use of the innovation through a combined strategy of social marketing, education, role modeling, training, and other similar activities. Includes: Opinion leaders Implementation leaders Champions External change agents Key stakeholders Innovation participants	Unclear whether leaders of this initiative were primarily external (B.K.Kee staff) or internal (CHDN staff) to the organization. Once the intervention was begun, all seemed supportive, though some more actively so than others. Degree of support appeared to correlate to some degree to degree of education of CHWs and therefore to their understanding of the process and intervention.
C. Executing	Definition: Carrying out or accomplishing the implementation according to plan.	Intervention implementation was successfully carried out despite barriers identified. Monitoring and evaluation of the intervention, another key deliverable, was more challenging with limitations in data quality, accuracy, and completion.
D. Reflecting & Evaluating	Definition: Quantitative and qualitative feedback about the progress and quality of implementation accompanied with regular personal and team debriefing about progress and experience.	Thoroughly done through quantitative data collection and in depth interviewing of workers responsible for implementation. All findings were fed back to implementing and funding organizations.

Researchers mapped implementation themes from interviews, observations, and examination of monitoring materials onto the CFIR constructs, as illustrated in Table 3. The most salient findings and themes about barriers of implementation are discussed in more depth below [25].

With respect to the initial training sessions, analysis of participant observation field notes resulted in three unique themes related to implementation barriers. The first related to an inner setting or culture CFIR domain. The idea of informed consent appeared to be a new, unclear, and unfamiliar concept to participants in the context of clinical care and as part of the research project. Asking for explicit consent was not part of normal clinic process. Participants felt that the process of obtaining informed consent would be intimidating for clients and health care providers. Physical consent sheets were provided as a tool to help guide the process, but these were also described as intimidating and CHWs preferred verbal consent to minimize patient concerns. Based on this feedback, shorter verbal consent was obtained instead of written consent.

The second theme from the training activities related to implementation barriers was in the process or planning domain. The data collection tools that had been developed prior to the training were not adequate. Due to project timing constraints, local CHDN staff had not had sufficient opportunity to inform tool development prior to training. To be functional, data collection tools required more collaborative development and a better understanding of local clinic contexts ideally with input from front-line CHWs. Tools were translated into Burmese, but the translation proved to be in language that was too complicated for participants (mostly whose first language was Kayah instead of Burmese). Tools were extensively edited during the training process with participant involvement and feedback to improve usability, specifically around a preference for close-ended categorical variables in data collection rather than open-ended free text.

A third kind of implementation barrier that emerged as a theme in participant observation field notes from the training activities was also in the process or planning domain. There were limitations in the training structure and ease of its implementation. The initial training session was mostly didactic in nature with the larger group, then breaking up into smaller groups to practice. However, it became evident that there were socio-cultural and hierarchal groups and separations between trainees impeding some participants from participating actively in training. Hierarchy was present at multiple levels with CHWs (of varying ages and levels of experience), CHDN administration staff, B.K.Kee funders, and researchers from Queen’s University all occupying potentially different places on that hierarchy. There was hesitancy in asking clarification questions by the CHWs because of the complex hierarchies involved. Language provided another layer of stratification. Training was partially in delivered in English (with simultaneous translations to Burmese) and partially delivered in Burmese. However, the local CHWs operate in a variety of dialects of Kayah, the local languages spoken in Karenni state. Although Burmese is the assumed lingua franca, comprehension of Burmese was a barrier in the training. It was unclear during the training session itself if all concepts were transferred effectively and this likely contributed to the need for ongoing support and retraining by CHDN staff during the early phases of implementation.

### Implementation

Implementation data were collected 9 months after initial training. From the nine interviews, five common themes emerged regarding barriers around implementation. These themes were supported and triangulated methodologically through analysis of participant-observer notes that were taken during or just following the clinic field visits.

#### CFIR mapping

With respect to the CFIR innovation characteristics domain, difficulties in vaccine transportation and logistics due to the external environment were noted in our study. The remoteness of some clinics made logistics difficult. For one clinic, the estimated travel time from the CHDN central office was four hours one way by motorcycle during the dry season. Women mainly delivered at home, which meant the program had to rely on women to contact the clinic CHWs to arrange for vaccination within the recommended 24-hour time frame. Some clinics were only accessible for certain parts of the year due to the rainy season making travel on dirt roads very difficult. Logistical issues were compounded by the fact that clinics did not have electricity or refrigerators to store vaccines, and some had only intermittent cellphone coverage.

*“*… *At first we planned for the refrigerator placing*… *we can locate the 6 refrigerators in the 6 clinics*. *Only 4 clinics have to transport from the central office*. *They can arrange for the electricity in some way*. *But in the actual*, *…[they] can’t do it*. *We provided all 6 refrigerators*. *Yes*, *we provide already*, *but they can’t locate it [to the clinics]*. *And*, *they only use the transportation [of the vaccine] from the central office till [now] so far*. *And till now*, *so it is a it is not very much matched with the planned activities*. *“*

–*B*.*K*.*Kee Manager*

Because of the lack of electricity and refrigeration, clinic CHWs had to arrange for vaccines to be delivered from the CHDN central office in the provincial capital on a case by case basis. Field visits confirmed the logistical challenges. Roads to even the closest clinic took two hours each way by truck with much of the road unpaved. All field visits were completed during the dry season, which meant that roads were still passable. But given that a majority of the roads were unpaved, it is very likely that many areas would be difficult to access during the rainy season. Continuous electricity was not available, although small solar panels were present and generators were occasionally used. As a result, none of the clinics were fully electrified meaning that it was not possible to maintain vaccine refrigeration on-site.

Another theme in the CFIR outer setting domain (cosmopolitanism) was related to some noted tensions in relationships between CHDN, CHWs and other health care stakeholders. Given that Karenni State is a conflict-affected zone, local ethnic health organizations such as CHDN sometimes have tense relationships with the central Burmese government healthcare officials. Initially, limited communication between CHDN and B.K.Kee Foundation staff with the central government regarding the HBV vaccination program led to tense interactions between CHDN CHWs and governmental physicians and midwives. This created a problem in delivering HBV vaccines to governmental hospitals if women enrolled in the program delivered in those facilities instead of CHDN clinics. There was a purported lack of trust from Burmese officials in the ability of ethnic health organizations to provide the correct standard of care due to the limited communication about HBV vaccination protocols between ethnic health organizations and Burmese officials. Further dialogue with the governmental health workers after this barrier was uncovered helped smooth out these relationships.

*“This is one of the challenges involved because they haven’t done any advocacy [with] the government about this [Hepatitis B] vaccination program so they have to deal with the government also about giving awareness*…*”*

–*CHDN Manager*

Observations during field visits also identified complex relationships between the different stakeholders as an important theme. Government clinics were present, but the buildings did not appear to be reliably staffed or in use. Despite supposed country-wide coverage delivered by the government, most of the infrastructure and governance in these remote regions were provided by CHDN and its affiliate organizations.

An unintended consequence of this program, also relevant to the CFIR outer setting (needs and resources) domain, was the tensions caused by significant community awareness raising around HBV infection, but limited HBV screening and vaccination for the wider community. The funded HBV vaccination program was available only for newborns of HBV positive women. However, part of the objectives of the program was community education as evidenced by over 1900 people attending information sessions about HBV. Because of the increased awareness of HBV as a health issue, CHWs expressed concern about the lack of treatment options available for HBV positive mothers and the lack of screening and immunization for other community members.

*“Because the hepatitis program is targeted mainly [to] the pregnant woman*, *other age groups*, *[spouses] and other people they also want to be tested and want to be vaccinated*, *and they ask [us] whether they can get it or not…but we have to say no*.*”*

–*Clinic CHW*

Awareness raising was successful in that community members were independently seeking information about screening for HBV. However, this may have also increased anxiety for community members, since there were no screening, treatment or vaccinations programs for those who could not afford to pay out-of-pocket for these services.

Concerns about quality of monitoring and evaluation data were also identified as a CFIR process domain theme during the interviews. In addition to program implementation, another deliverable of this project was capacity building around appropriate program monitoring and evaluation. There were initial concerns with data accuracy, translation, and completion. The tools provided were used regularly, but with inconsistencies in the data when reassessed by CHDN managers.

*“So regarding the monitoring the 10 clinics what he is seeing is that there are some data inconsistencies between the self reporting so he is trying as much as he can he also calls them by phone and sees if the data is correct or not*.*”*

–*CHDN Manager*

Field visits to the clinics nine months post-implementation confirmed variable record-keeping quality. Records were filled, but not all elements relevant to this project were necessarily completed. Yet extensive data was captured in medical visit logs regarding demographics, chief complaints, and routine treatments, providing evidence for ability and proficiency in maintaining records when deemed important.

Suboptimal data quality likely resulted from a need for more training, reflecting a CFIR inner setting domain (readiness for implementation). The clinic CHWs stated that they would have appreciated more monitoring and evaluation training and refreshers throughout the program. They also wished that more clinic CHWs had attended the initial training instead of having only one lead CHW per clinic attend and then train other CHWs in the respective clinics.

*“There were no pamphlets for this program*. *She insists on giving refresher trainings if should they expand the program–refresher trainings/new trainings*. *Also invite all of these participants that is in charge of this and by all of them*, *not the focal person only*. *“*

–*Clinic CHW*

This was supported by the initial field visits to several clinics whereby B.K.Kee staff had to review training again with CHDN clinic staff. The reasons and importance of good and complete data quality may not have been conveyed adequately during the training sessions.

#### Strengths

Although data collection was focused on barriers and challenges in implementation, several strengths were apparent in both observations and through the interviews. Themes were mostly grouped in the inner setting domains concerning networks and communication and readiness for implementation. Many interviewees commented on the support by the CHDN central office in implementation of the program. Despite the logistical challenges, communication within the organization was not a barrier as vaccines were able to be shipped to the remote clinics in a timely manner.

*“There is not much difficulty with this program*. *Because they are engaged with the central [CHDN] office in Loikaw*. *Should they need anything they can ask [the CHDN manager] for transportation*, *transportation of the vaccines and tests*.*”*

–*Clinic CHW*

Interviewees identified the strong leadership of CHDN central office staff in providing support for the program in areas of re-training and troubleshooting any issues that arose. This was also apparent during observations during field visits whereby CHDN managers assisted in the data collection process and appeared to be responsive to clinic CHW needs.

## Discussion

Our results indicate the feasibility of a vaccination program for remote, ethnic minority communities in a resource limited setting in Myanmar. The program was successful in preventing HBV vertical transmission in HBV positive mothers with no vaccinated infants testing positive for HBsAg. This is consistent with modelling that suggests that controlled temperature chain outreach strategy for delivery of HBV birth dose vaccine can reduce the burden of HBV associated with perinatal infection [27]. This is particularly important in the context of the February 2021 military coup which has led to a disintegration of the country’s healthcare system and is likely to further marginalize ethnic minority groups in Eastern Myanmar and elsewhere.

It is interesting that the HBV prevalence in the current study was lower compared to other studies. On the Thai-Burmese border, 8.5% of women were HBsAg positive whereas in this study only 4.6% were positive [4]. Our prevalence is also lower than the 6.5% estimated for Myanmar as a whole [3]. Further research is warranted to determine if there are any systematic differences between subgroups to explain different HBV prevalence or whether the differences are due to sampling bias.

It is noteworthy that no vaccinated infants tested positive for HBV. Without vaccination, we expect 4–36 newborns to be positive for HBsAg, given a vertical transmission rate of 10–90% depending on relative HBsAg and HBeAg positivity in the mothers [6]. Importantly, this was only assessed through a highly sensitive and specific point of care HBsAg test, and not confirmed through gold standard serology, allowing for the possibility of false negatives and misestimates of initial HBV prevalence among pregnant women. The study was also completed prior to the formal recommendations for third trimester antivirals [11] therefore further study is required regarding the implementation and effectiveness of antiviral provision in this setting. Nevertheless, this study adds to the body of evidence supporting the effectiveness of HBV vaccination alone in preventing vertical transmission. The current estimates of PMTCT effectiveness are also higher than those reported for a HBV vaccination program in rural India [14].

This study’s qualitative results around implementation are a unique strength. To our knowledge, no current published studies describe the implementation factors and considerations in such a resource limited rural setting or in the context of Myanmar. A similar study describes barriers to immunization among Burmese migrant workers living in Thailand, but it does not discuss the in-country Myanmar context nor does it use an implementation framework [28]. This study adds to the literature in describing potential barriers and considerations for successful program implementation both in this specific setting as well as resource-poor settings and in conflict affected settings more generally.

CFIR has been used in the past to analyze implementation of immunization programs both in developed and developing countries [29, 30]. Similar to other immunization programs, we identified barriers in multiple domains. However, unlike other studies, the identified concerns in this setting were not consistently spread among all implementation domains. Instead, most concerns were focused on the outer setting and within the process group of constructs. There were few perceived barriers concerning the innovation itself such as the feasibility or validity of the program or an individual’s ability to implement. Interestingly, participants did not identify challenges in recruiting women to participate in the program, nor challenges in immunizing or testing neonates. While stigma around hepatitis B infection might have been a limitation, this was not identified by participants, rather they were unable to meet the demand for hepatitis B testing due to limited resources. Although a major barrier was adaptability of the innovation to the resource limited setting, the program was well accepted by all participants. The concern about logistical issues was readily overcome by CHDN. The program may have been so well accepted due to a local understanding that immunization is well within the scope of healthcare delivery for CHDN. Alternatively, acceptance may have been higher due to the positionality of the researchers in comparison to the participants. Because of the power differential, certain themes may be hidden during the research process [31].

Identified barriers emphasized the importance of process and understanding the outer setting. There were various barriers associated with the planning and execution phases of the process such as issues with adapting training materials to local needs and the monitoring and evaluation requirements of the program. These barriers could be easily mitigated through longer term development of the tools as well as early involvement of stakeholders and end users to ensure that the tools are appropriate and useful. The overall program was designed and implemented quickly over a few months and would have benefitted from a longer planning phase and training period to overcome these process barriers. Unforeseen issues such as consent not being part of normal health care could then have been identified and addressed in advance. In future partnerships, a longer planning phase and earlier engagement with the local stakeholders is recommended.

The outer setting barriers echo the complex environment of Karenni state and the importance of adapting programs to the local context. The friction between governmental and non-governmental stakeholders was not fully anticipated by project leads. Additionally, further pre-planning would have been useful to assess the needs of the community and to address unintended consequences such as increasing stress in the community about HBV without the ability to offer broader testing and treatment. A needs assessment would have been beneficial to both mitigate the unintended consequences and to set priorities in such a resource limited setting [32, 33].

The program in Karenni State provides a model for collaborative efforts in vaccination program implementation in a resource limited setting. The incorporation of the local ethnic health organization, CHDN, likely explains the success of this program. Mapping data onto the CFIR, it became apparent that the inner setting domains were sources of strength that moderated implementation barriers. There were positive structural characteristics with a combination of stability and resources in the partnership between CHDN and B.K.Kee. CHDN also had effective internal processes that fostered positive communications and readiness for implementation. It appeared that most of the barriers identified were able to be overcome due to the commitment and capacity of CHDN. Despite the complex environment, CHDN was also able to adapt to the changing circumstances and to support their staff throughout implementation. Issues such as the lack of electricity to power vaccine refrigerators and complicated relationship issues between CHDN and the government were overcome. Having local stakeholder involvement allowed for a community based participatory approach to implementation, which is associated with more effective and sustainable interventions [34].

The successful implementation of this program is also a hopeful step in this conflict-affected setting. CHDN was able to navigate the relationship between Burmese government officials and overcome this barrier to improve overall health in the region. At the time of this study, it was hoped that the ability to work parallel to the Myanmar government might provide a roadmap of peace through health in the region. Obviously, the landscape has changed dramatically since the military coup of February 2021. Nevertheless, it may be that further, potentially collaborative, efforts centred around health and led more by Burman and ethnic health workers rather than governmental agents or policy could enhance the relationship leading to beneficial effects to the local population. Reaching an understanding about superordinate health goals can be a mechanism that is further developed to achieve lasting peace [35].

Strengths of this study include the multiple modalities of capturing the implementation experience and contextual factors. The mixed methodology incorporating multiple data collection and analytical approaches allowed for increased credibility of the results and increased depth of analysis into barriers. This approach enabled analysis on both the process and the outcome, which are key to implementation success.

Limitations of this study include the lack of a control group to reliably assess vaccine effectiveness. The before-after nature of the study only allowed estimation of vaccine effectiveness based on assumptions of baseline HBV positivity. Furthermore, exact maternal HBV status with viral loads and HBeAg testing was unable to be completed due to the resource limited setting therefore clouding understanding of possible reasons of why this regimen was effective. Additionally, monitoring was only done at discrete time points by the study team without continuous embedment during the study period. With already existing issues in data management, data collection inaccuracies could have affected the results. Language and cultural barriers were a significant issue in interpretation of the findings. However, the incorporation of multiple perspectives mitigated potential problems through triangulation of the themes.

## Conclusion

Taken together, the results show that a complicated vaccination program is feasible and effective in a resource limited rural region in Myanmar without government health system involvement. Despite the complexities of the setting, which have only increased recently barriers were able to be overcome. Ethnic health organizations have demonstrated their innovation and creativity for decades in the context of waxing and waning conflict with the central government^9^. This vaccination campaign was successful in preventing HBV vertical transmission, with no infants testing positive for HBsAg. Further longitudinal research is needed to assess the sustainability of this intervention and its potential expansion to other immunizing agents.

## Abbreviations

CFIR: Consolidated Framework for Implementation Research
CHDN: Civil Health and Development Network
CHW: Community Health Workers
HBeAg: Hepatitis B e Antigen
HBIG: Hepatitis B Immunoglobulin
HBsAg: Hepatitis B Surface Antigen
HBV: Hepatitis B Virus
PMTCT: Prevention of Maternal to Child Transmission
WHO: World Health Organization
